# Effect of Mortise and Tenon Structure on the Properties of Wood Flour Polyvinyl Chloride-Laminated Veneer Lumber Co-Extruded Composites

**DOI:** 10.3390/polym15092151

**Published:** 2023-04-30

**Authors:** Guanggong Zong, Jinjiang Zhou, Mengyan Zhang, Yanqiu Ma, Yang Zhao, Xiaoyan He, Jianxiu Hao, Fangfang Wang

**Affiliations:** 1 College of Fine Arts and Design, Yangzhou University, 88 Daxue South Road, Yangzhou 225009, China; 2 Suzhou Crownhomes Co., Ltd., 289 Shijin Road, Suzhou 215000, China; 3 Research Institute of Zhejiang University, Taizhou 318000, China; 4 School of Chemistry and Chemical Engineering, Suzhou University, Suzhou Education Park, Suzhou 234000, China

**Keywords:** co-extruded composite, mortise and tenon combination, wood–plastic, laminated veneer lumber, interface bonding, creep resistance

## Abstract

Core–shell composites with strong weather resistance, mechanical strength and creep resistance can be prepared using co-extrusion technology. Considering the weak bonding strength between core–shell interfaces, this study started from the concept of a mortise and tenon combination; three types of conical, rectangular and trapezoidal mortise and tenon joints were prepared, and their bending properties, long-term creep properties, interfacial bonding properties, and dimensional stability properties were tested. Results showed that the mortise and tenon structure could form a mechanical interlock between the outer-shell-layer polyvinyl chloride (PVC) wood–plastic composite (WPVC) and the inner-core-layer laminated veneer lumber (LVL), which could effectively improve the interface bonding property between the two layers. Among them, the trapezoidal mortise and tenon structure had the largest interface bonding force compared with the tapered and rectangular mortise and tenon structure, where the interface bonding strength reached 1.01 MPa. Excellent interface bonding can effectively transfer and disperse stress, so the trapezoidal mortise and tenon structure had the best bending properties and creep resistance, with a bending strength of 59.54 MPa and a bending modulus of 5.56 GPa. In the long-term creep test, the deformation was also the smallest at about 0.2%, and its bending properties, creep resistance and interface bonding performance were also the best. The bending strength was 59.54 MPa and the bending modulus was 5.56 GPa; in the long-term creep test, the strain curve was the lowest, about 0.2%. In addition, the mortise and tenon structure could disperse the stress of the inner shell LVL after water absorption and expansion, thus significantly improving the dimensional stability of the co-extruded composite after water absorption.

## 1. Introduction

Wood-flour-reinforced polymer composite (WPC) is an environmentally friendly material used in both the forestry and plastic industry [1,2,3]. WPC has advantages in terms of low cost, recyclability, water resistance, acid resistance, alkali resistance, and termite resistance [4,5]. In recent years, it has been widely used in transportation, interior decoration, landscape, and other fields [6,7,8]; however, some performance defects have been gradually exposed with the application and promotion of WPC. For example, the creep property of resin matrixes makes WPC products creep and creep-rupture under long-term load, which greatly limits the application of WPC in load-bearing structures [9,10,11].

There are many factors affecting the creep resistance of WPC, such as fiber type, content, length–diameter ratio, orientation distribution, melt index of the matrix, interface compatibility, etc. Therefore, the researchers also conducted a modification exploration on these aspects to improve the mechanical properties of WPC, including creep resistance [12,13,14,15,16]. Considering the economic cost, environmental benefits, and operability, we prefer physical combinations to improve the creep resistance of composites. Research has shown that the combination of materials with good creep resistance can effectively improve the creep resistance of WPC [17,18].

Laminated veneer lumber (LVL) is a kind of engineering wood bonded by veneer, which is usually used in structural and nonstructural applications, such as flooring, furniture, and architecture [19,20]. Compared with solid wood, LVL has better dimensional stability and higher elastic and rupture moduli due to its production technology that can disperse the defects of wood nodes [21,22]; however, LVL can easily absorb and desorb water from the environment due to the hygroscopicity of wood. This cycle destroys the dimensional stability of LVL and promotes its degradation and failure.

A composite combining the performance characteristics of WPC and LVL, i.e., strong weather resistance, high mechanical strength, and strong creep resistance can be prepared by co-extrusion molding technology. This technology involves the use of two or more extruders to continuously extrude two-phase or multiple materials into multilayer composite materials through specific molds [23,24,25]. At present, co-extrusion technology has been applied to the production and preparation of WPC composites, namely Co-WPCs. Generally, Co-WPCs have a core–shell structure, which provides a platform for the design of Co-WPCs with different properties to adapt to different use conditions [26,27]. However, the core–shell interface bonding ability of co-extruded composites is poor, and stress cannot be effectively transferred between interfaces, which greatly limits the synergistic effect between the shell and core layers.

In this study, poplar LVL was used as the core layer and polyvinyl chloride (PVC) wood–plastic composite (WPVC) as the shell layer to prepare Co-WPCs with a core–shell structure; however, the interface between them was easy to peel off due to their different response to temperature and humidity. Considering this technical difficulty, this study started from the concept of a mortise and tenon combination to mill the groove of poplar LVL in the core layer and changing the combination mode of the two at the physical structure level to achieve a more powerful physical combination. This study aimed to explore the influence of the milling groove shape (conical, rectangle, and trapezoid) on the interface bonding properties of the two; compare the mechanical properties, creep properties, water absorption properties, and dimensional stability of Co-WPCs under the same milling area; and provide new ideas and methods for expanding the preparation technology and application fields of Co-WPCs.

## 2. Materials and Methods

### 2.1. Materials

Poplar veneer was purchased from Harbin Yongxu Co., Ltd., Haerbin, China. Powdered poplar veneer was purified to make it pass through an 80-mesh screen. PVC resin, with model S700 and polymerization degree 700, was purchased from Qingdao Bay Chemical Co., Ltd. (Qingdao, China). Poplar LVL, with a density of 0.66 g cm^–3^ and size 74 × 35 × 2000 mm^3^, was purchased from Yelu Wood Industry Co., Ltd. (Dezhou, Shandong, China). A 2.2 to 2.6 mm thick rotary cut poplar veneer with 250 g m^−2^ single-sided urea formaldehyde resin adhesive was used for preparing the poplar LVL. The veneers were assembled using horizontal parallel grain lapping and then prepressed at 1.2 MPa for 40 min, followed by surface repairing. Then, the assembled veneers were further hot pressed at 1.5 MPa for 20 min and surface-repaired. After equilibrium, the prepared LVL was cut according to the customized size and sanded. Calcium–zinc stabilizer was purchased from Ancheng Chemical Co., Ltd. (Qingdao, China). Industrial-grade calcium stearate was purchased from Shandong Gaomi Youqiang Additive Co., Ltd. (Qingdao, China). Industrial-grade polyethylene wax and stearic acid were purchased from Harbin Yongchang Chemical Products Distribution Office (Harbin, China).

### 2.2. Preparation of Composites 

#### 2.2.1. Preparation of WPVC

The shell layer WPVC was prepared as depicted in Table 1. The mixtures were mixed using an 80 kg high- and low-speed mixer. The formula powder that was stirred was granulated using a 97/132 twin-screw granulator, and the granulation processing parameters were as presented in Table 2. (The processing temperature is mainly determined by the molding temperature of PVC and the empirical value of our laboratory).

#### 2.2.2. Groove Milling on Poplar LVL

**Figure 1:**
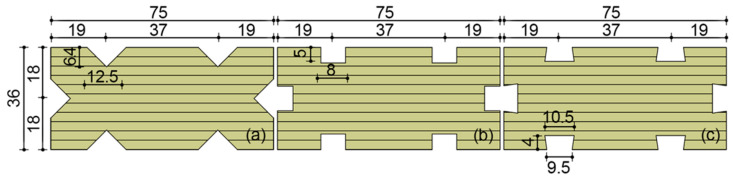
Different shapes of LVL: (**a**) conical; (**b**) rectangular; and (**c**) tarpezoidal.

Planer and double-sided press-planer equipment were used to process poplar LVL squarely in advance to ensure the accuracy of poplar LVL dimensions with different milling groove shapes after processing. During slot milling, a fixed processing guiding rule was set at 19 mm from the slot milling tabletop to the centerline of the router. According to the depth and quantity requirements of the core material slot milling scheme, the corresponding surfaces of poplar LVL were milled using vertical drilling and milling machine equipment. The groove milling cutters with different shapes were processed according to the depth marked in the design scheme. The milling section area of each notch was 40 mm^2^, and the total milling area was 240 mm^2^ so as to avoid errors in the mechanical properties of poplar LVL with different processing shapes due to different milling areas, thus affecting the differences in mechanical properties of the prepared co-extrusion composite materials. The processing shapes of different milling grooves of poplar LVL are shown in Figure 1. The poplar LVL without milling grooves and that processed with conical, rectangular, or trapezoidal milling grooves were named as OLVL, conical-milled groove LVL sample (CLVL), rectangular-milled groove LVL sample (RLVL), and trapezoidal-milled groove LVL sample (TLVL), respectively.

#### 2.2.3. Preparation of WPVC–LVL Wood–Plastic Co-Extrusion Composites

**Figure 2:**
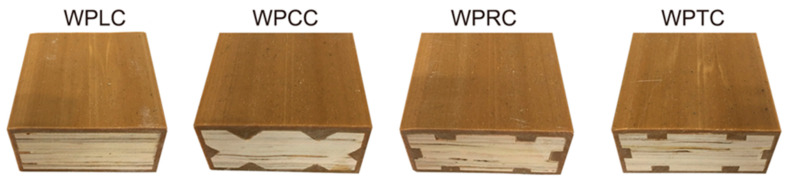
Cross-section of WPVC-LVL co-extruded composite.

The WPVC–LVL co-extrusion composite material was prepared using a 65/132 cone twin-screw co-extrusion extruder. The processing parameter settings of the twin-screw co-extrusion extruder are depicted in Table 3, and a section of the sample is shown in Figure 2. The prepared samples were sawed into corresponding sizes according to the size requirements of each test method. Before testing, the sample ports were sealed with a preservative film and placed in a dry and closed environment. The co-extrusion composite materials prepared using unmilled LVL and conical-, rectangular-, and trapezoid-milled LVL were named WPLC, WPCC, WPRC, and WPTC, respectively.

## 3. Characterization Methods

### 3.1. Bending Performance

**Figure 3:**
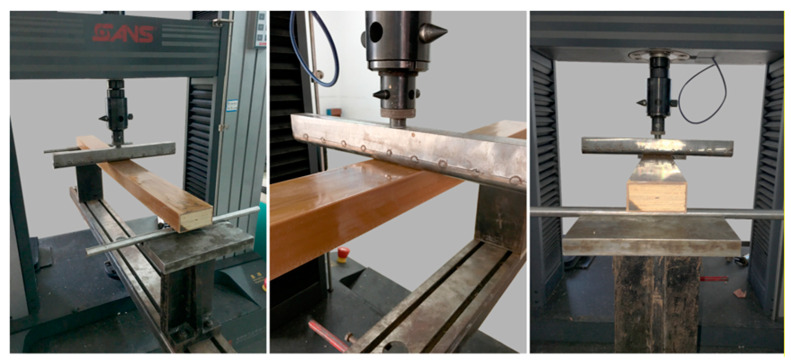
Bending property test of WPVC-LVL co-extruded composites.

The WPVC–LVL co-extruded composite was prepared to a specification of 950 × 80 × 41 mm^3^ as per the requirements for three-point bending test samples based on the American Society for Testing and Materials (ASTM) D790 standard. The ratio of span to thickness was 20:1, which was 820 mm. The test method is shown in Figure 3. The experimental equipment was RGT-20A universal mechanical testing machine (CMT5504, MTS Shenzhen, China). As the thickness of the sample was 41 mm, a 15 mm diameter backup roll and a 30 mm diameter loading roll were used. The loading speed was 5 mm min^–1^. Each group of experiments was repeated five times, and the average value was taken.

### 3.2. Long-Term Creep Performance 

**Figure 4:**
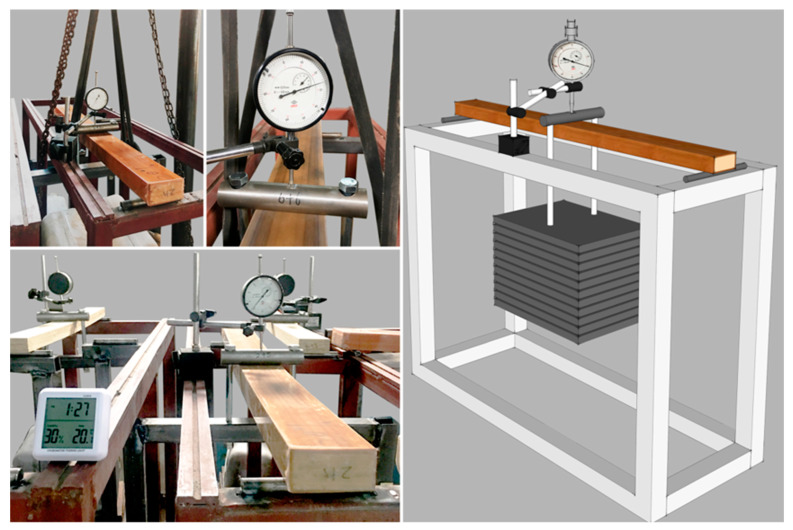
Testing of three-point bending creep properties of WPVC-LVL co-extruded composites.

The size of the WPVC–LVL co-extruded composite was 950 × 80 × 41 mm^3^, and the ends of the sample were sealed with epoxy resin adhesive. At room temperature, the constant load applied during the creep test was determined to be 150 kg according to the average peak bending force of the sample. The test method is shown in Figure 4.

The creep test cycle was 1500 h, and the two groups of data were recorded. The corresponding creep strain values were calculated based on the ASTM D2900 standard.

The transformation between strain and deformation was calculated using the equation:

(1) $$\varepsilon =6Dd/L^{2}$$

where ε is the strain (mm/mm); *D* is the deformation (mm), that is, the difference between the corresponding dial indicator reading and the initial reading at time *t*; *L* represents span (mm); and *d* is thickness of the sample (mm).

### 3.3. Interface Bonding Performance

The interface bond strength between the core layer poplar LVL and the WPVC shell layer was tested using the standard GB/T 17657-2013 interface bond strength test method. The rectangular sample was of a size 50 × 50 × 25 mm^3^ with a milled circular area of 1000 mm^2^. Six samples were used for each group. The mechanical properties were tested using the RGT-20A (Nanjing, China) universal mechanical testing machine with a test loading rate of 2 mm s^−1^. The samples were loaded uniformly during the test, and the maximum failure load value was recorded. After the test, the failure surface was photographed to observe the pullout morphology of the interface.

### 3.4. Dimensional Stability 

#### 3.4.1. Water Absorption Expansion Test

The WPVC–LVL co-extrusion composite samples were cut into square samples measuring 80 × 80 mm^2^ (length × width). The initial weight and the thickness of each sample were tested after being dried at 50 °C to reach the mass balance. The samples were immersed in a water tank with a pH value of 7 and a temperature of 20 °C ± 2 °C. The water temperature remained unchanged during the test. The surface of the sample was perpendicular to the water surface and soaked for 168 h until it was damaged. The expansion values of nine points at the fixed position of the width surface and nine points at the fixed position of the thickness surface of the sample were tested every 12 h after wiping the surface moisture. The average volume expansion rate of the sample was calculated using the values of the nine points after calculating the expansion rate of each point. 

#### 3.4.2. Water Absorption Weight Gain

The cyclic-tested samples were taken out of the water, and the water on the surface was wiped out. Then, the sample was weighed using an analytical balance. The weight gain from water absorption in each stage was calculated based on the original weight. The test was conducted in 10 min on samples taken out of the water after each cycle. 

#### 3.4.3. Morphological Analysis after Water Absorption

During the water absorption expansion test, the photographs of each group of samples were taken from the initial stage to the final stage of the test. The main factors that affected the corresponding morphology of each group of samples in different stages were summarized, recorded, and analyzed.

## 4. Results and Discussion

### 4.1. Bending Performance Analysis

**Figure 5:**
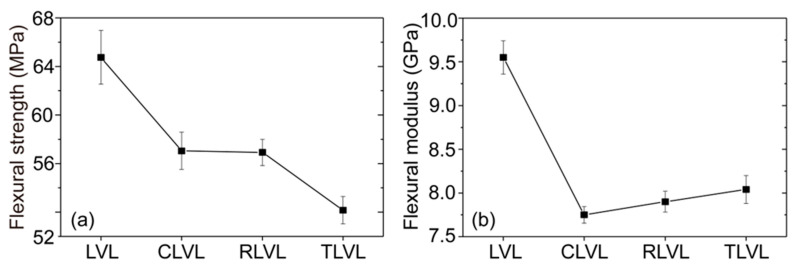
The bending properties of LVLs with different shapes: (**a**) bending strength and (**b**) bending modulus.

As shown in Figure 5a, the bending strength of the OLVL was 64.75 MPa. The bending strength of the CLVL was similar to that of the RLVL, which was 57.05 and 56.92 MPa, respectively. Compared to the OLVL, the bending strength decreased by about 12.00%. The bending strength of TLVL was the lowest among the four groups of samples, which was 52.17 MPa with a decrease of 19%. The slotting process reduced the volume of LVL by 8.90%; at the same time, it destroyed the structure of the LVL, leading to a decrease in the bending strength of the LVL. However, the decrease in the bending strength of LVL was different from the difference in milling groove shapes. This was because the top of the sample bore the pressure, and the bottom generated tensile force when the sample was subjected to bending. The different milling groove shapes led to different stress distribution of the sample, resulting in different degrees of reduction.

Besides the bending strength, milling groove processing also led to a decrease in the bending modulus of the LVL. As shown in Figure 5b, the bending modulus of the OLVL was 9.55 GPa, and the bending modulus of the RLVL and TLVL was 7.90 and 8.04 GPa, respectively. Compared to the OLVL, the bending modulus decreased by 17.28% and 15.80%, respectively. The bending modulus of the CLVL sample was the lowest (7.75 GPa), with a decrease of 18.8%. The effect of milling groove treatment on the LVL bending modulus was greater than on the bending strength. Further, the width of the groove-milling rabbet of the CLVL, RLVL, and TLVL samples was 12.5, 8, and 9.5 mm, respectively, based on the comparative analysis of the dimensions of the three milling-groove shapes, and the depth of the milling grooves was 6.4, 5, and 4 mm, respectively. When the samples were subjected to bending deformation under pressure, the stress distribution expanded from the surface to the interior of the samples. The size and depth of the groove milling rabbet of the RLVL and TLVL were smaller than those of the CLVL. Consequently, the RLVC and TLVL samples were able to withstand and disperse more destructive forces when stressed.

**Figure 6:**
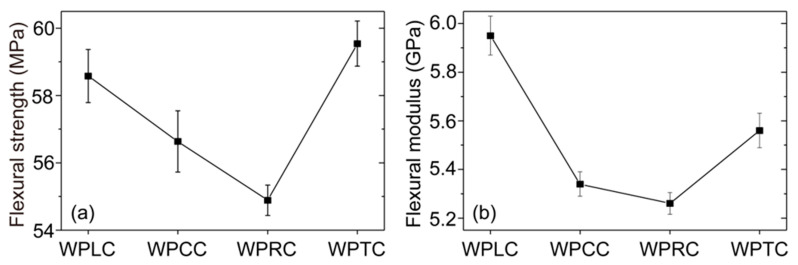
Bending properties of WPVC-LVL co-extruded composites: (**a**) bending strength and (**b**) bending modulus.

LVL could be covered effectively with WPVC using co-extrusion technology. LVL grooves were filled with WPVC under the pressure of the extruder and mold. The co-extrusion composites with different mortise and tenon structures could be obtained after cooling and shaping. Compared with WPLC co-extrusion sample, the bending strength of the co-extrusion sample WPCC and the WPRC decreased to varying degrees (Figure 6a). The bending strength of the WPLC sample was 58.58 MPa, and the bending strength of WPCC and WPRC was 56.64 and 54.89 MPa, respectively; however, the bending strength of the WPTC co-extrusion sample was 59.54 MPa, which was higher than that of WPLC,WPCC, and WPRC. The bending modulus followed a similar trend with the change in bending strength. The bending modulus of the WPRC sample was the lowest (5.26 GPa) and that of the WPTC sample was the highest (5.56 GPa) among the co-extrusion composites with the three types of mortise and tenon structures (Figure 6b). This result might be due to two reasons: First, when the co-extrusion composite was fractured due to the destructive force, the difference strain of the outer shell layer WPVC and the inner-core-layer LVL resulted in LVL damage. The milling process led to the reduction in LVL volume and structural damage, resulting in the lower bending strength and modulus of the co-extrusion composite with mortise and tenon structures than that of the WPLC. Second, different mortise and tenon structures had different effects on the bonding between the outer-shell-layer WPVC and the inner-core-layer LVL. The conical and rectangular mortise and tenon structures could increase the contact surface between shell and core layer, improve the friction force, and thus increase the binding force, but this kind of structure had poor constraint ability. The trapezoidal structure with narrow upper and wide lower areas made the outer shell layer WPVC and the inner-core-layer LVL form a strong mechanical interlock, which was capable of achieving effective stress transmission when stressed, so that it had high bending strength and modulus.

### 4.2. Analysis of Long-Term Creep Performance 

**Figure 7:**
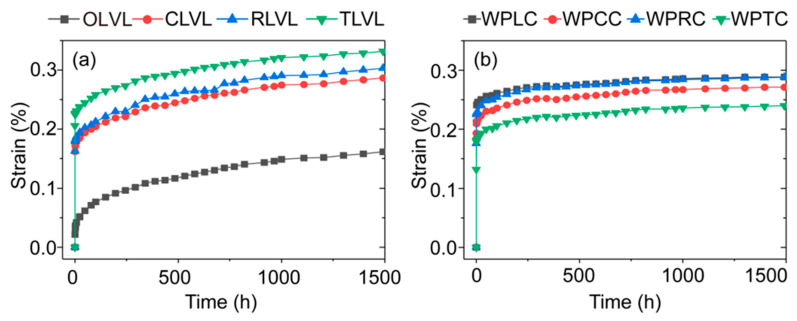
Long-term creep properties of LVLs (**a**) and WPVC-LVL co-extruded composites (**b**).

Figure 7 shows the strain–time curve of LVL and WPVC–LVL co-extruded composites. The figure shows that the strain increased gradually with the increase in time. The curve shows two obvious stages: elastic and viscoelastic. For all LVLs, the transition time from the elastic stage to the viscoelastic stability stage was relatively long. Under the same load and duration, the strain level of OLVL was the minimum, the strain of CLVL was close to that of RLVL, and the strain of TLVL was the maximum. The change rule was the same as that of the bending strength. The main reasons were volume reduction and structural damage of the LVL. The WPVC–LVL co-extruded composite sample showed a more stable viscoelastic stage than the LVL sample (Figure 7b). In the whole test time range, the strain curves of WPLC and WPRC almost coincided, the strain curve of WPCC decreased significantly, and the strain curve of WPTC was the lowest, which was the result of the influence of different mortise and tenon structures. Among these, a better combination of the outer shell layer WPVC and the inner core layer LVL could be obtained using the trapezoidal mortise and tenon structure. This is because when the co-extruded composite was under long-term stress, the creep resistance of the shell layer WPVC was poor, while the creep resistance of the core layer LVL was better. The resulting strain difference was effectively controlled by the mechanical interlocking mechanism formed by the trapezoidal mortise and tenon structure. The creep resistance of the shell layer and the core layer was complementary and balanced, so the creep resistance increased.

### 4.3. Analysis of Interface Bonding Performance

**Figure 8:**
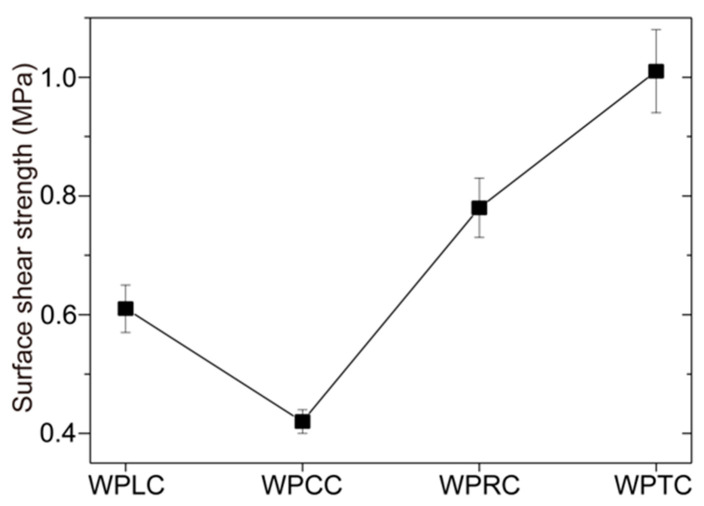
Core-shell interface binding strength of WPVC-LVL co-extruded composites.

**Figure 9:**
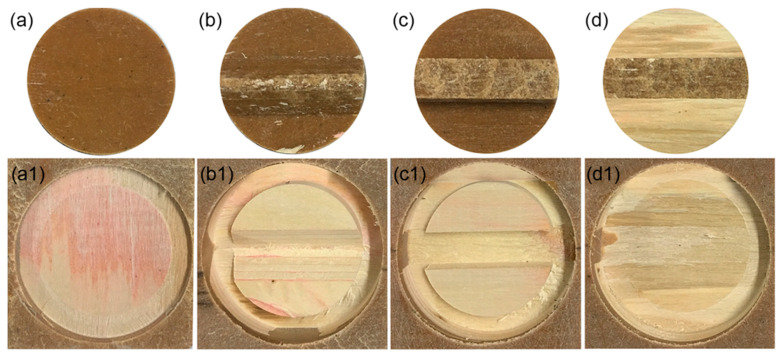
Core-shell interface separation morphology of WPVC-LVL co-extruded composites: (**a**) shell of WPLC; (**a1**) core of WPLC; (**b**) shell of WPLC; **(b1**) core of WPCC; (**c**) shell of WPRC; (**c1**) core of WPRC; (**d**) shell of WPTC; (**d1**) core of WPTC.

The interface bonding properties between the outer shell and the inner core was different with the different mortise and tenon structures of the WPVC–LVL co-extrusion composites. The interface bonding strength of the WPCC sample was the lowest (0.42 MPa) (Figure 8). The separation interface between the outer shell and the inner core was relatively smooth (Figure 9b,b1). Hence, it could be concluded that the tapered mortise and tenon structure did not effectively improve the interface bonding strength. The interface bonding strength of WPLC sample was slightly increased to 0.61 MPa (Figure 8), and the separation interface was also smooth (Figure 9a,a1). However, the interfacial bonding strength of WPRC and WPTC samples improved significantly, which was 0.78 and 1.01 MPa, respectively (Figure 8), and their pullout interfaces were rough (Figure 9c,c1,d,d1). This indicated that the interfacial bonding strength could be improved effectively by rectangular and trapezoidal mortise and tenon structures, but the trapezoidal mortise and tenon structure exhibited the best strength. The shell and core layers interlocked effectively due to the large bottom area of trapezoidal structure and small area of the top surface. It was necessary to overcome larger constraint force to cause interface bonding failure when pulling out. Residual wood fibers existed on the pullout interface of the WPTC sample because the mechanical bonding force between WPVC and LVL exceeded the interlaminar bonding strength of LVL (Figure 9d,d1).

### 4.4. Analysis of Dimensional Stability 

**Figure 10:**
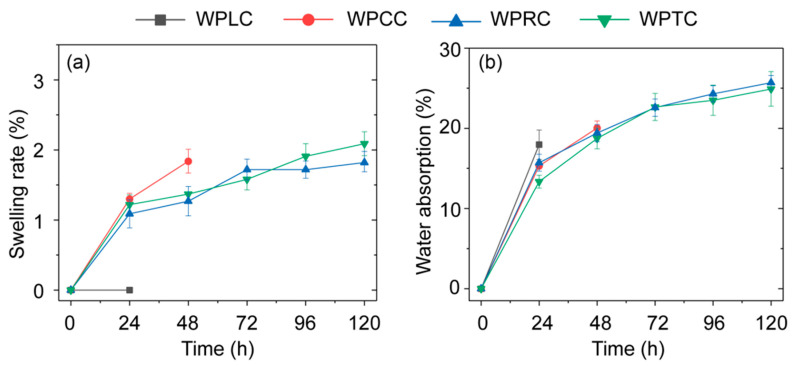
Water absorption property of WPVC-LCL co-extruded composites: (**a**) swelling rate and (**b**) water absorption.

**Figure 11:**
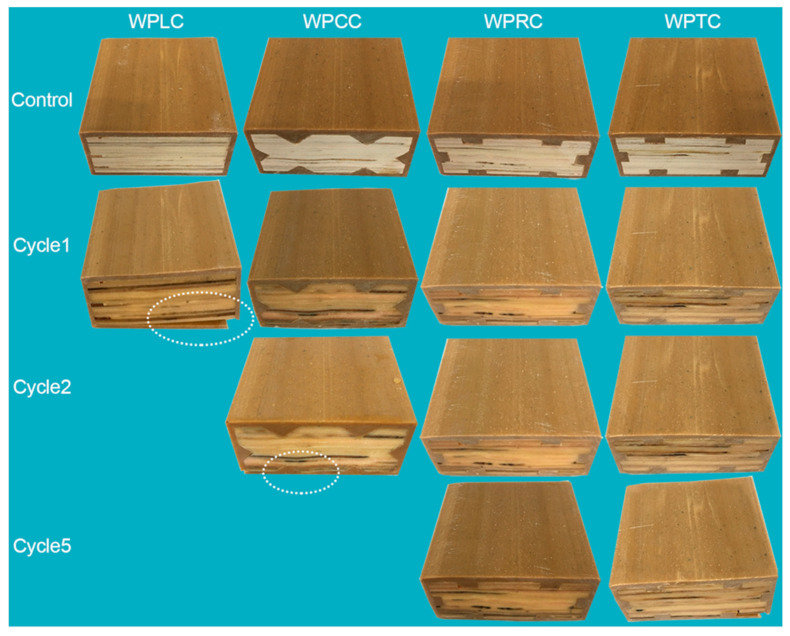
Appearance of co-extruded composites after absorbing water.

Water is one of the main environmental factors affecting material properties, especially for poplar LVL with its hydrophilicity. Generally, poplar LVL can easily absorb water and expand, which affects its dimensional stability and results in the reduction or even the loss of its mechanical properties. The presence of WPVC in the outer layer can reduce the water absorption of LVL and also limit the water absorption expansion of the core LVL. The water absorption expansion rate and the water absorption weight gain rate both increased with the increase in time (Figure 10). After the first 24 h cycle test, the water absorption weight gain rate of the WPLC sample was the highest and the shell cracking occurred (Figure 11), causing the termination of the experiment. The co-extrusion composite with mortise and tenon structures remained intact. At the end of the second 24 h (total 48 h) cycle test, the WPCC shell broke and the water-absorption weight gain reached the maximum level. After the fifth 24 h (total 120 h) cycle test, the WPTC sample also broke, and only the WPRC sample was in good shape (Figure 11). Compared with WPLC, the co-extrusion composite with mortise and tenon structures had obvious advantages in terms of inhibiting water absorption expansion. This was because of the decrease in LVL volume after milling and thus the decrease in water absorption and volume expansion rate. Further, WPVC of the outer shell layer could disperse the destructive force generated by water absorption and wood expansion through the mechanical inlaying of notches when LVL of the inner core layer absorbed water for expansion, thus maintaining the dimensional stability of composite materials. In addition, for trapezoidal mortise and tenon structure, its internal could form mechanical interlocking. When the core layer LVL absorbed water and expanded, the shell layer WPVC had poor water absorption and could not expand synchronously with the core layer. Due to mechanical interlocking, the expansion stress of the core layer would be dispersed by the shell layer, thus effectively limiting the water absorption deformation of the core layer.

## 5. Conclusions

In this study, the co-extrusion composite materials with three types of mortise and tenon structures were prepared based on the concept of mortise and tenon structures. The bending properties, creep resistance, interface bonding properties, and dimensional stability were tested and analyzed. The conclusions were as follows:

(1) The bending properties of LVL decreased significantly due to the decrease in the volume of poplar LVL in the core layer and the damage to the structure during milling. The co-extrusion of milled LVL and WPVC could effectively compensate for the performance degradation caused by LVL milling. The bending performance of WPTC exceeded that of WPLC. At the same time, based on the effective reinforcement of mortise and tenon structure, the creep performance of milled LVL and WPVC after co-extrusion for 1500 h improved significantly.
(2) The mortise and tenon structure could provide greater bonding stress to inhibit the separation of the two layers when they were pulled out. Among these, the trapezoidal mortise and tenon structure could form the mechanical interlock, which had the largest interface bonding force compared with the conical and rectangular mortise and tenon structures, and had more advantages in realizing the physical bonding between the outer shell layer and the inner core layer.
(3) The coating of the outer shell layer WPVC on the inner shell layer LVL could slow down the water absorption by the LVL. The milling process reduced the volume of the LVL, which could reduce the water absorption capacity of the sample. Moreover, the mechanical interlocking formed by trapezoidal mortise and tenon structure could disperse the stress of the core layer LVL after water absorption and expansion, thus significantly improving the dimensional stability of the co-extrusion composites.

In this study, we wanted to expand the application of WPC in civil engineering by relying on the performance of LVL; however, we increased the core–shell bonding force at the expense of the mechanical properties of LVL, resulting in unsatisfactory results. In addition, even with the same design concept, the properties of the material itself limit its application field. Compared with artificial fibers, such as glass fibers [28,29], natural fibers have lower strength, which is also a key factor for its low comprehensive mechanical properties. However, the co-extruded composites we studied can be used in the field of light weight with low mechanical requirements, such as gazebos, sunrooms, etc.

## Figures and Tables

**Table 1:** WPVC of different wood fiber content (wt.%).

Samples	PVC (wt%)	Wood Flour (wt%)	Calcium–Zinc Stabilizer (wt%)	ACR (wt%)	PE Wax (wt%)	Stearic Acid (wt%)	Calcium Stearate (wt%)
WPVC	100	40	6	4	0.6	0.4	0.3

**Table 2:** Process parameters of WPVC pellet.

Heating Process	Temperature of Different Heating Stages (°C)								Feed Speed (kg h^−1^)	Extruded Velocity (rpm)
	1	2	3	4	5	6	7	Handpiece		
Twin-screw granulator	150	160	170	180	180	170	170	170	3	55

**Table 3:** Process parameters of WPLCs.

Heating Process	Temperature of Different Heating Stages (°C)								Feed Speed (kg h^−1^)	Extruded Velocity (rpm)
	1	2	3	4	5	6	7	Mold		
Co-extruder	165	170	175	178	178	178	170	180	45	60

## Data Availability

The data used to support the findings of this study are available from the first author upon request.
